# Animal protein toxins: origins and therapeutic applications

**DOI:** 10.1007/s41048-018-0067-x

**Published:** 2018-10-11

**Authors:** Na Chen, Siqi Xu, Yuhan Zhang, Feng Wang

**Affiliations:** Laboratory of Protein and Peptide Drugs, Institute of Biophysics, Chinese Academy of Sciences, Beijing, 100101 China

**Keywords:** Animal venoms, Protein and peptides, Targets, Human diseases, Clinical applications

## Abstract

Venomous animals on the earth have been found to be valuable resources for the development of therapeutics. Enzymatic and non-enzymatic proteins and peptides are the major components of animal venoms, many of which can target various ion channels, receptors, and membrane transporters. Compared to traditional small molecule drugs, natural proteins and peptides exhibit higher specificity and potency to their targets. In this review, we summarize the varieties and characteristics of toxins from a few representative venomous animals, and describe the components and applications of animal toxins as potential drug candidates in the treatment of human diseases, including cancer, neurodegenerative diseases, cardiovascular diseases, neuropathic pain, as well as autoimmune diseases. In the meantime, there are many obstacles to translate new toxin discovery to their clinical applications. The challenges, strategies, and perspectives in the development of the protein toxin-based drugs are discussed as well.

## INTRODUCTION

**Fig. 1:**
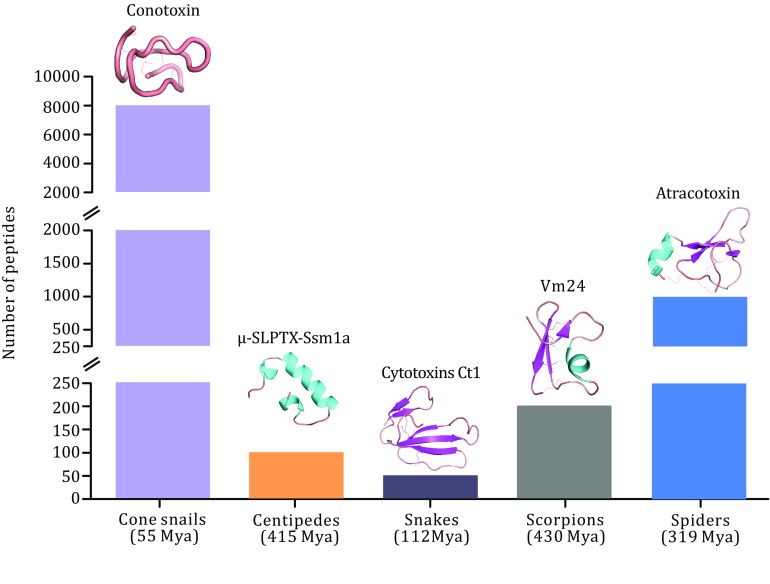
Comparison of the numbers of unique peptides in different venomous animals and the presentation of promising drug candidates identified from their venoms

Animal venoms are composed of varieties of proteins and peptides fine-tuned by millions of years of evolution. These toxins target multiple ion channels, receptors, and enzymes with high potency and sometimes exquisite selectivity, therefore, attracting much attention to further investigate their pharmacological and physiological properties. Toxins synthesized by venomous animals from both terrestrial animals and marine animals, such as scorpions, snakes, spiders, bees, cone snails, and sea anemones, are injected into the body for hunt or defense by animal wounding apparatus, such as fangs, barbs, spines, and stingers. Some venomous animals have been used to treat diseases for millennia in many parts of the world. Scorpion, as an example, has been used to treat spasms and endogenous wind in traditional Chinese medicine. Many important effects have been discovered when studying the functionality of animal toxins. The high selectivity and potency make animal toxins as brilliant pharmacological tools and drug candidates. Proteins and peptides are the main active components in animal venoms (Fig. 1), which mostly contain multiple disulfide bonds. The numbers of disulfide bonds vary in different venomous species, such as 10–40 cysteine residues have been found in cone snails, 40–80 residues in scorpions and snakes, with remarkable structural diversities resulted from long-term selective pressure and coevolution process. Although protein toxins show multiple advantages compared to small molecule drugs for therapeutic applications, such as higher potency and selectivity, they still face many challenges, such as the short circulating half-life, the lack of membrane permeability, and poor oral bioavailability as well. In this paper, we reviewed the resources of venomous animals, the representative components of their venoms, and the applications in treatment of human diseases. Finally, we discussed the challenges and opportunities of the drugs derived from natural animal toxins.

## THE SOURCES OF ANIMAL VENOMS

Here, we only describe the popular toxins from Kingdom Animalia. The composition of venoms depends on the species. In many cases, a small dose of them can lead to painful disorders or even death. The common and well-known venomous animals include genus *Conus* (cone snails), arthropods (spiders, scorpions, centipedes, bees, *etc*.), vertebrates (snakes, lizards, *etc*.), and cnidarians (jellyfishes, sea anemones, *etc*.) as well.

### Venoms from genus *conus* (cone snails)

**Fig. 2:**
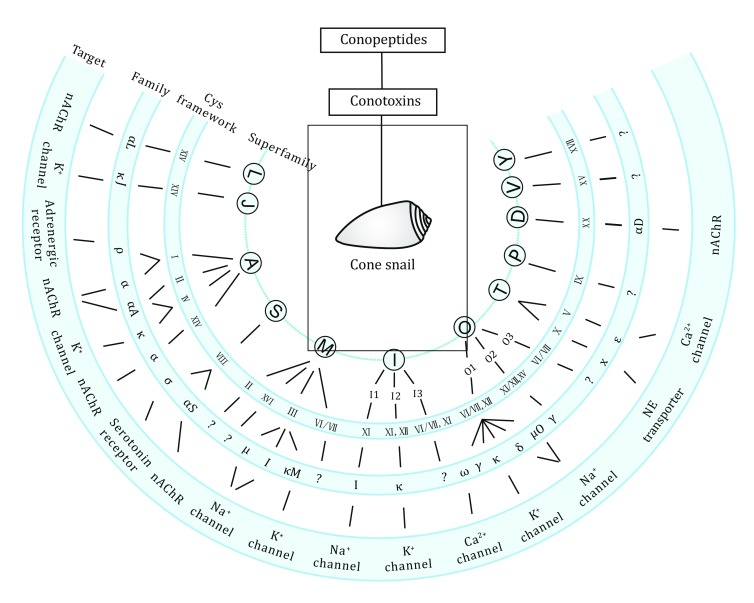
An example of the classification of disulfide-rich conotoxins modified from Akondi’s work (2014). Based on the homology of their conserved signal sequence, cysteine frameworks, as well as the targets, conotoxins can be classified into numbers of superfamilies and families. NE: norepinephrine; nAChR: nicotinic acetylcholine receptor

The Conoidea superfamily (cone snails, *etc*.) is an extremely diverse group of predatory marine neogastropoda divided into 16 families, among which, the cone snails are the most extensively studied. The toxins produced by venomous snails are mostly bioactive peptides targeting the ion channels or receptors in the nervous systems. They are generally 10–30 amino acids in length and rarely more than 60, with high specificity and affinity to their targets. More than 80,000 conotoxins so far have been found in various cone snails all over the world. Cone snail toxins named as conotoxins or conopeptides are generally classified by three major criteria. We categorized the toxins in Fig. 2 according to Akondi’s work (2014). Various conotoxins are the natural ligands to many ion channels including Na^+^ channel, K^+^ channel and Ca^2+^ channel, receptors, such as nicotinic acetylcholine receptor, serotonin receptor, and transporters. ω-MVIIA (ziconotide), approved by the U.S. Food and Drug Administration (FDA), is the most famous product derived from cone snail venom, which displays remarkable analgesic activity in treating chronic pain via its potent inhibitory effect on N-type Ca^2+^ channels (Rigo *et al*. 2013; Sahand *et al*. 2016).

### Venoms of snakes

More than 200 species of venomous snakes exist in the world. Four major venomous snake families include *Hydrophiae*, *Elapidae*, *Viperidae*, and *Crotalidae* (Matsui *et al*. 2000). Snake venom is the combination of proteins, peptides, inorganic ions, and so on, with specific chemical and biologic activities. Meanwhile, it seemed that the relatively common and similar components of venoms are produced by the same family of snakes, but they are basically different depending on each snake species (Ouyang *et al*. 1990; Tu 1996). For example, many neurotoxins and dendrotoxins have been found dominantly in *Elapids* and *Hydrophids*, while myotoxins and hemorrhagic toxins are frequently discovered in venoms of *Crotalidae* and *Viperidae*. As neurotoxins, cytotoxins, cardiotoxins, nerve growth factors, various enzymes (*e.g.,* proteases, phospholipases), enzyme inhibitors, *etc*., some components of snake venoms can be used as pharmacological or diagnostic tools, and even drugs to treat cancer, chronic pain, neuromuscular disorders, autoimmune diseases, and blood and cardiovascular disorders. (Calderon *et al*. 2014; Pal *et al*. 2002; Wang and Qin 2018).

### Venoms of scorpions

There are thirteen families and about 1750 scorpion species in the world. Generally, the smaller scorpion species with slender claws have more toxic venoms. Scorpion venom is a mixture of compounds containing salts, nucleotides, biogenic amines, enzymes such as phospholipase, hyaluronidase, L-amino acid oxidase, metalloproteinase, serine protease, mucoproteins, as well as small peptides. Some of these venoms have been well studied, laying foundation for further drug development effort in the pharmaceutical industry. For example, components of scorpion venoms possess anticancer activities. The anticancer effects are achieved mainly via regulating the expression of ion channels, *e.g.*, Cl^−^ channels, K^+^ channels, and Na^+^ channels, inhibiting cell proliferation or inducing apoptotic activities by modulation of cell cycle or activation of caspase-dependent apoptosis pathways (Cohen-Inbar and Zaaroor 2016; Ding *et al*. 2014a; Wang and Guo 2015). Furthermore, the components of scorpion toxin have antimicrobial activities. Three types of antimicrobial peptides have been identified according to the structural homology. The details of biological activities and mechanism of actions of the antimicrobial peptides from scorpion venoms have been well studied and reviewed by Harrison *et al*. (2014).

### Venoms of spiders

Spiders are the overwhelming majority of venomous animals on the planet. The number of spider species is predicted to be about 150,000, which is perhaps greater than the total numbers of all other venomous creatures. Yet, we only have limited knowledge on spider species and their venoms. Spider venoms are complex containing active peptides proteins, and low molecular mass compounds, which target a large group of receptors, ion channels, and enzymes. Most of known disulfide-rich peptides and proteins isolated from spider venoms usually target to excitable cell membranes, primarily modulating the functions of ion channels (Ca^2+^, Na^+^, and K^+^), membrane receptors, and so on. So far, only a small number of the spider venoms have been identified and pharmacologically characterized, but their biological activities are potent and impressive. Spider toxin peptides exert many biological functions containing neuroprotective effect (Chassagnon *et al*. 2017), antimicrobial activity (Samy *et al*. 2017), analgesic effect (Deuis *et al*. 2017), enzyme inhibitory activities, *etc*. For instance, ASIC1a, a subunit of acid sensing ion channels (ASICs) is a novel therapeutic target for many pathophysiological diseases, such as ischemia, pain, neurodegenerative diseases, autoimmune disorders, and mental diseases. (Chen and Wang 2016; Deval and Lingueglia 2015; Wang *et al*. 2016b; Zeng *et al*. 2014). So far, the only potent and specific inhibitor of ASIC1a is π-TRTX-Pc1a, a 40-residue peptide isolated from spider venom (Cristofori-Armstrong and Rash 2017).

### Venoms of centipedes

Centipedes are one of the oldest extant venomous predators on the planet, and there are about 3500 species all over the world. Centipedes have been used as traditional Chinese medicinal animal for hundreds of years to treat many disorders including children’s convulsions, hemiplegia, epilepsy, cough, scrofula, tuberculosis, cardiovascular diseases, and so on. It was also found that the decoction of centipedes has anti-inflammatory effects and analgesic activities. Unlike scorpions and spiders, relatively less attention was drawn to centipede venoms due to their small body size and low venom yields in the past. Recently, lots of studies indicate that the components of centipedes are composed of many antimicrobial peptides, cytotoxic enzymes, ion channel modulators, protease, and neurotoxin peptides as well (Hakim *et al*. 2015), which could provide valuable resource for drug discovery. For instance, components from Chinese red-headed centipedes, *S. subspinipes mutilans,* have been identified as modulators of voltage-gated ion channels (Yang *et al*. 2012, 2013), which makes centipedes venoms as potential drug candidates for pain and other channelopathy. Another novel peptide, colopendin 1, was also isolated from *S. subspinipes mutilans*, which showed potent antimicrobial activity with minimal side effects (Choi *et al*. 2014). Subsequently, more than 17 novel antimicrobial peptides have been found from *S. subspinipes mutilans*. Their antimicrobial activities have been evaluated by *in vitro* and/or *in vivo* models. Among them, ten synthetic peptides were identified for their potent antimicrobial activities with no toxic effect to erythrocytes in mouse model (Fratini *et al*. 2017), which makes them valid alternatives to traditional small molecular weight antibiotics.

## THE THERAPEUTIC APPLICATIONS OF ANIMAL TOXINS IN DISEASES

Animal venoms are complex mixtures. Some components in mixtures can potently and selectively act on the protein targets and show pharmacological effects to relevant human diseases and disorders. The components of venoms from various species have been analyzed in many studies. The toxins across the species often have low sequence homology, and different structural characteristics. Interestingly, some toxin peptides or proteins from different species act on the same target, but the effects of those toxins may be different dependent on their specificities and inhibitory activities toward the target proteins. Those toxins with high specificity, high potency, and proper inhibitory activity are the basis of drug design. In following paragraphs, toxins with reported activities or applications in various diseases are discussed.

### Anticancer activity

Cancer is a disease induced by some reasons including genetically or environmentally caused mutations, infections with pathogens, bad living habits, drug abuse, *etc.*, leading to uncontrolled division of abnormal cells in a part of the body, which directly affects human living quality and even life. Many treatments have been adopted to prevent the growth of cancerous cells or to remove precancerous tissue that may turn into cancer. Surgery procedures are usually used together with the treatment of chemotherapy and/or radiation therapy. The chemotherapy is based on the administration of small molecular weight chemicals to kill cancerous cells, commonly lacking of target specificity, taking more risks of undesired side effects, such as bone marrow toxicity, cardiotoxicity, and immunosuppression leading to enhanced risks of infection. Antitumor drug development based on natural animal venoms has become one of the new strategies to handle these problems.

In general, toxins from natural resources exhibit potent cytotoxic activity to cancerous cells via modulation of the apoptosis response pathway, impairment of cancer proliferation, inhibition of enzymatic activities, or alteration of the cell cycle. Among those, snake venoms are rich source of cytotoxins for oncological studies. Cytotoxins (CT1, CT2, and CT3), lectins, metalloproteinases, aggretin, disintegrins, and rhodostomin are components identified from different type of snake venoms, some of them have been verified the validity on the treatment of various human cancers (Macedo *et al*. 2015; Calderon *et al*. 2014; Jain and Kumar 2012; Vyas *et al*. 2013).

It was found that altered or abnormal expression of ion channels on the surface of cells are involved in cancer processes and pathology. Therefore, the animal venoms targeting ion channels (including Na^+^, K^+^, Ca^2+^, and Cl^−^) may have selective activities against cancer cells. Anticancer effects of animal venoms have been studied cross several species, including bee, scorpion, spiders, and so on, targeting ion channels in different cancer types. For example, AGAP peptide from BmK scorpion venom acting as the selective inhibitor of a voltage-gated sodium channel was verified to have antitumor activity due to its role in the impairment of cell cycle and interfering with factors essential for modulating signaling pathways relevant to cell survival and growth, apoptosis, cell proliferation, and so on (Zhao *et al*. 2011). Chlorotoxin and other scorpion toxins associated with ion channels also have anticancer potentialities (Ding *et al*. 2014b). Moreover, venom components from other animal species, like family *Bufonidae*, bees, and spiders, are also found antitumor activities (Mahadevappa *et al*. 2017; Oršolić 2012; Rodriguez *et al*. 2017).

### Animal venoms for the treatment of neurodegenerative diseases

Neurodegeneration is the progressive disease resulting in the loss of structures or functions, and the final lethal destiny of neurons. Neurodegenerative diseases including Parkinson’s disease (PD), Alzheimer’s disease (AD), Huntington’s disease, epilepsy, multiple sclerosis, amyotrophic lateral sclerosis, *etc*., affect millions of individuals worldwide. So far, such diseases are incurable and there is no particularly effective drug available for treatment, making an urgent need for effective drugs exploring. Neurodegeneration can be triggered by genetic mutation, protein misfolding, oxidative stress, mitochondrial damage, neuro-inflammation, glutamatergic excitotoxicity, aging, aggregate formation, and apoptosis. Because humans have been part of the long-term coevolution process together with prey and predator animals, some animal venom components exhibit excellent selectivity and affinity for various targets located in human central nervous systems. Recent research indicates that animal venoms are rich in neuroactive molecules, which can be pharmacological drug tools to analyze the pathological progress of neurodegenerative disorders and provide good candidates for new drug development. For instance, snake venoms rich in neuroactive components have been widely studied for its potential therapeutic applications in neurodegenerative disorders, such as AD. The dendrotoxins extracted from the venom of the African mambas (*Dendroaspis* genus) could block K^+^ channel, and increase acetylcholine release, therefore, useful to treat AD induced by acetylcholine deficits. Metalloproteases from snake venom can degrade the amyloid β-protein (Aβ), one of the components of amyloid plaques, in an animal AD model. (Nalivaeva *et al*. 2012). Bee venom from *Apis mellifera* possesses the anti-inflammatory effect, and can reduce microglial activation, CD4+ T cells infiltration, and oxidative stress as well as improve motor coordination and balance in various animal Parkinson’s models (Awad *et al*. 2017; de Souza *et al*. 2018; Silva *et al*. 2015).

### Analgesic activity

**Fig. 3:**
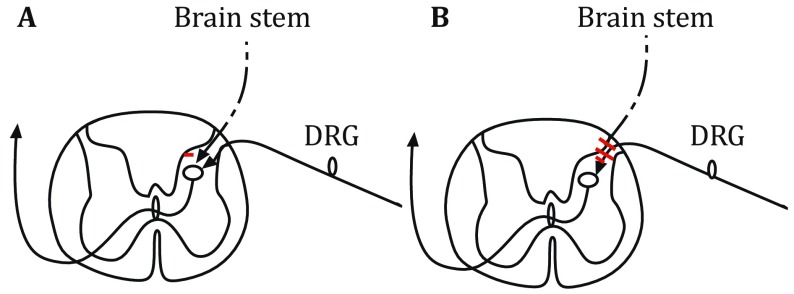
Descending pain-inhibitory systems in spinal cord. Diagrams **A** and **B** illustrate two different ways for brain stem descending axons to inhibit pain signaling from nociceptive primary afferent nerve fibers in spinal cord. **A** Direct postsynaptic inhibition of pain signaling. **B** Indirect presynaptic inhibition of pain signaling via volume transmission of an inhibitory neurotransmitter released from brain stem descending inhibitory axons

Neuropathic pain is a chronic pain disease, caused by neuron damages or firing aberrantly (Hamad *et al*. 2018). During the process of pain signaling delivery in neurons, postsynaptic neurons are activated or inhibited by excitatory or inhibitory neurotransmitters released from presynaptic neurons, respectively (Fig. 3). Many channels and transporters contribute to the neurotransmitter release or Ca^2+^ fluxes, and they are the targets of toxins, which are allowed to modulate the neuron signaling delivery. Among them, toxins having high specificity and selectivity on the targets can be used as drug candidates. For example, ω-conotoxin MVII A (25 amino acids) from *Cone* snail is the blocker of N-type Ca_V_2.2 voltage-gated calcium channel (VGCC) (Adams and Berecki 2013), which is located on central terminals of primary sensory dorsal root ganglion (DRG) neurons (nociceptors) in spinal cord (Cizkova *et al*. 2002). The blockage of Ca_V_2.2 channel in presynaptic neurons inhibits the action potential formation in the secondary pain sensory neuron and stops the pain signaling delivery. Because of the high potency of ω-conotoxin MVII A, its synthetic version (ziconotide) has been applied to treat neuropathic pain as a medicine called Prialt. Also, the Chi-conotoxin MrIA, isolated from *Conus marmoreus*, is a noncompetitive inhibitor of the norepinephrine transporter (NET) on a terminal of brain stem descending axons. The blockage of NET in presynaptic membrane by Chi-conotoxin MrIA stops the reuptake of neurotransmitter noradrenaline, hence increasing the volume of noradrenaline in the synaptic cleft. Noradrenaline, a transmitter released from descending axon terminals, can block the propagation of pain signals by two different ways, (1) inhibition of the activity of spinal relay neurons by hyperpolarizing cell membrane potentials (a postsynaptic inhibition); (2) suppression of the terminal activities of nociceptive primary afferent fibers through volume transmission of noradrenaline (a presynaptic inhibition), therefore preventing the generation of action potential and blocking the ascending pain signals (Pertovaara and Almeida 2006). Toxin-derived drugs for the treatment of neuropathic pain are usually tested *in vivo* by using mouse allodynia model (Nielsen *et al*. 2005) and hypersensitivity (Obata *et al*. 2005) behavioral assays. The synthetic version of Chi-conotoxin MrIA, Xen2174 (13 amino acids) (Sharpe *et al*. 2003), has been tested in clinical phase II for the treatment of neuropathic pain.

### Cardiovascular diseases

Cardiovascular disease is a class of diseases that involve the heart and blood vessels, such as hypertensive heart disease, heart failure, stroke, thromboembolic disease, and so on. There are several examples of venom-based drugs for the successful treatment of cardiovascular diseases. Bradykinin-potentiating peptide b (BPPb), which is from the Cantil snake venom, is a well-known toxin for the decrease of blood pressure, where the toxin targets an enzyme on tissue surfaces, and is the blocker of human angiotensin-l converting enzyme (ACE). According to renin-angiotensin systems, ACE located on the external surface of endothelial cells of lungs and kidneys is the essential enzyme for generating angiotensin II (AngII), therefore leading to the increase of blood pressure (Masuyer *et al*. 2012). The enzyme ACE also carries out the effect on cleavage of bradykinin which is a vasodilator for decreasing the blood pressure. Thus, the inhibited ACE fails to produce AngII (a vasoconstrictor) and cleave bradykinin, finally resulting in the decrease of blood pressure. Capoten (captopril), the small molecule drug designed according to structure–activity studies of BPPs, has been widely used for the treatment of hypertension. However, this medicine could have many side effects caused by the free thiol group in the captopril molecular structure. Hence, optimized structural forms of peptide BPPs would improve the treatment of hypertension with reduced side effects.

Venoms are also found to be useful antithrombotics. Thrombosis is the obstruction of the blood vessels through the circulatory system due to the blood clot formation inside the vessels. A key event of thrombus formation is platelet aggregation (Scarborough *et al*. 1991). Platelets aggregate and adhere to collagen in the sub-endothelial layer of blood vessels through the binding of its receptors (*e.g.,* integrins) with adhesive proteins, such as fibrinogen and von Willebrand factor (VWF). Platelets are then triggered by the binding of collagen, leading to downstream kinase cascades and intra-platelet signaling. In the meantime, the platelet receptor glycoprotein (GP)IIb-IIIa, which is a member of a superfamily of integrins (Scarborough *et al*. 1991), is converted to their active form to initiate platelet aggregation. The peptide barbourin, from the venom of the southeastern pigmy rattlesnake, *Sistrurus m. barbouri*, is identified to specifically inhibit the binding of adhesive proteins on the integrin GPIIb-IIIa during the receptor-mediated platelet aggregation (Scarborough *et al*. 1991), therefore the peptide barbourin is termed “disintegrin.” There are other venom peptides, such as tergeminin, echistatin, and eristicophin, working as “disintegrin” to inhibit the binding of adhesive proteins to integrins (Scarborough *et al*. 1991). Researchers are convinced that the inhibitory function of disintegrins to platelet aggregation attributes to their anti-thrombotic effects (Lewis and Garcia 2003).

### Autoimmune diseases

Autoimmune disease is an abnormal condition arising from an abnormal immune response to a normal body part. Medications acting on the target with selectivity, specificity, and less side effects are needed. Previous studies found that in the process of T lymphocyte immune response, the voltage-gated potassium channel K_V_1.3 together with calcium-activated K^+^ channel (K_Ca_) contribute to the membrane potential and calcium signaling in T cells (Shen *et al*. 2017). The up-regulated expression of K_V_1.3 in T and B lymphocytes is observed during the development of autoimmune diseases (Wang *et al*. 2016a). Some toxins from scorpion venoms and sea anemone venoms are found to have the inhibitory functions on K_V_1.3 channel, which results in the deactivation of effector memory T (T_EM_) and the secretion of related cytokines, therefore reducing the symptoms of autoimmune diseases. Although many toxins can block K_V_1.3 channel, such as ShK, BmKTX, OSK1, HsTX1, and so on (Shen *et al*. 2017), only a few toxins can be utilized as therapeutics for autoimmune diseases according to their potencies, specificities, and inhibitory activities (*K*_d_ and *IC*_50,_ *EC*_50_) to this channel. The synthetic version of toxin ShK (ShK-186) (Wang *et al*. 2016a), produced with an unnatural amino acid, obtains improved specificity to K_V_1.3 channel and has been applied in clinical phase II (Shen *et al*. 2017). Apart from the improvement of toxins through synthetic versions, many studies focus on toxin-antibody fusion proteins as therapeutics. One previous study claimed that toxin Vm24 fused in CDR loop of an antibody presents excellent potency, specificity, and inhibitory activity (Wang *et al*. 2016a). Overall, animal toxins have brilliant prospective on the research and development of therapeutics in various diseases.

## CHALLENGES AND OPPORTUNITIES OF ANIMAL PROTEIN- AND PEPTIDE-BASED DRUGS

Most of the peptides and proteins from animal venom are natural ligands of membrane ion channels or receptors with exquisite specificity and high potency, thereby driving many interests to drug exploring. However, native peptides and proteins as drugs are still confronted with many challenges from discovery to clinical application. Firstly, it is highly difficult to isolate or study a single component of peptide or protein in a certain amount from extremely limited supplies of venom. Secondly, most of the toxin peptides are rich in disulfide bonds, the gene engineering and chemical synthesis remain expensive and uncertain to yield enough bioactive products with desired disulfide bridge, since the primary, secondary, and final tertiary structures of proteins and peptides are crucial to their specificity and functionality. Thirdly, the short serum half-lives of many bioactive proteins and peptides limit their final efficacy to their targets in the treatment of diseases. In summary, the advantages and disadvantages are listed in Table 1.

**Table 1:** The advantages and disadvantages of natural peptides acting as drugs

Advantages	Disadvantages
High activity	Conformational instability during storage and transportation
High specificity	Potential immunogenicity
Low toxicity	Poor oral bioavailability
Broad targets	Short serum half-life
High biological diversity	Low membrane permeability
	Poor solubility

**Fig. 4:**
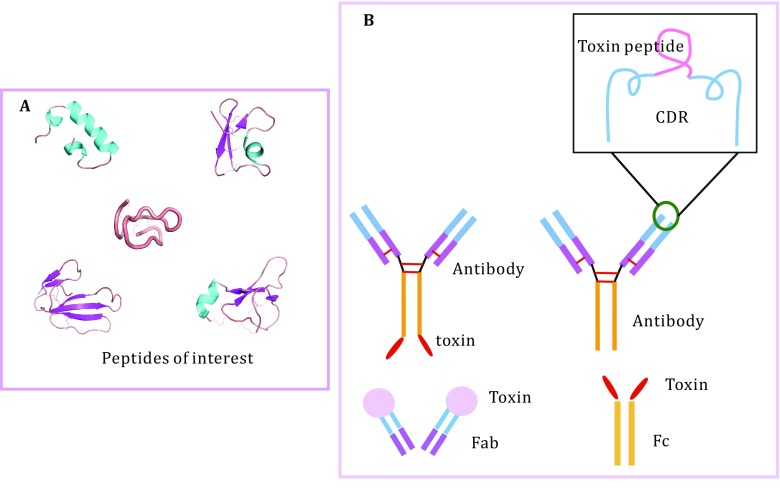
Toxin peptides of interest and toxin peptide–antibody fusion. **A** Toxin peptides of interest. **B** Toxin peptides–antibody fusion. Four patterns of fusion are illustrated: toxin–(Fab)Ab, toxin–(Fc)Ab, toxin–Fc, and toxin–Fab

The development of biotechnology methods enabled production of large quantities of bioactive proteins and peptides that are derived from components of animal venoms at a lower cost and in a relatively easier manner. Several strategies have been developed to extend half-life and reduce immunogenicity of the therapeutic proteins and peptides. These strategies include genetic manipulation of amino acids relevant to stability and/or immunogenicity, genetic fusion to proteins or protein domains, such as immunoglobulin, human serum albumin (HSA), antibody FC domain, as well as chemical conjugation to synthetic polymers, such as PEG. In addition, the development of new drug-delivery systems and new drug forms, such as microspheres, liposomes, and nano- or micro-particles, are also technological options to optimize pharmacological profiles of protein- and peptide-based drugs. Since the first description of a CD4-Fc fusion protein in 1989 (Capon *et al*. 1989), this class of engineered proteins has been widely adopted (Fig. 4). For instance, the scorpion toxins OsK1 and OdK2 have been successfully fused with antibody FC domain or human serum albumin without reduction of K_V_1.3-blocking activity (Edwards *et al*. 2014). While the counteraction of peptide-Fc fusions includes unexpected inflammatory responses, the reduction of biological activity, aggregations of some proteins, and so on. Human serum albumin is another widely used fusion protein vehicle. Compared to Fc fusion, recombinant HAS are technically easier and more efficient, but in most cases the specific activity of HSA-fused toxins has been affected by the presence of the albumin moiety. Chemical conjugation of small proteins and peptides with PEG extends the plasma half-life through increasing the hydrodynamic radius of a protein or peptides, but the PEGylation may reduce the affinity of peptides to its targets in some cases, *e.g*., PEGylation of an analog of Shk prolonged the plasma half-life dramatically, but decreased the affinity for K_V_1.3 (Murray *et al*. 2015). Furthermore, PEG is non-biodegradable, which may limit its usage in the development of protein- and peptide-based drugs.

Peptide toxins usually contain multiple disulfide bonds which are essential for the structural stability and bioactivity. Although, a combination of technologies has been employed to form the desired disulfide bridges, generating peptides with multiple disulfides are still a great challenge. We recently reported that the structure of BLV1H12, a bovine antibody, has an ultralong heavy-chain complementary determining region, CDR3H, which folds into a unique structural motif. The solvent anti-parallel β-strand stalk and the cross-link knob domain afford a unique “stalk-knob” structure, which was successfully used to graft a number of proteins and peptides to generate stable and potent functional bovine antibody-CDR3H fusions (Liu *et al*. 2014; Zhang *et al*. 2013a, b, 2014). Furthermore, Vm24, a natural immunosuppressive peptide collected from Mexican scorpion with high specificity to Kv1.3 channel, was grafted into the CDR regions of the humanized BVK and Synagis (Syn) antibodies by using β-sheet and coiled-coil linkers, respectively. The fusion protein Syn-Vm24-CDR3L exhibited excellent selectivity and potency to human effector memory T cells. The fusion antibody also demonstrated highly improved plasma half-life and stability in rats compared to the parent Vm24 peptide. The efficacy of the fusion protein was then tested in the rat delayed-type hypersensitivity (DTH) model. As expected, a significant suppressing effect has been detected. (Wang *et al*. 2016a). Moreover, fusion-site optimization and pharmacophore modification could be performed in these peptide-fusion antibodies to further increase the affinity, selectivity, and inhibitory activity to K_V_1.3 channel. Based on this new type of antibody skeleton, each natural protein and peptide from animal venom can be converted to a recombinant antibody with one or more functionalities, which may hold great promise for the development of new natural protein- and peptide-based drugs.

## PERSPECTIVES FOR THE FUTURE

As reported, the market of protein- and peptide-based drugs is growing up to $40 billion per year, or approximate 10% of the total ethical pharmaceutical market. Undoubtedly, we can imagine the number of peptides identified and clinical applications will continue to grow up quickly in future. The innovations for improving the pharmacological profile of protein/peptide-based drugs, such as new fusion approaches, novel chemical synthesis methods, new invention of drug-delivery systems, will become prevalent. Obviously, some key technical hurdles still need to be conquered for the development of effective protein- and peptide-based therapies, such as improvement of the membrane permeability and biological barriers transportation for the therapeutic proteins and peptides. Furthermore, having clear knowledge of therapeutic needs, the relevance of bioactive proteins and/or peptides to their membrane targets and the interaction between them is indispensable for the drug discovery and development. Finally, the rational design of natural protein- and peptide-based drugs with biological methods or in silico tools should be reinforced to accelerate the efficiency of screening and optimizing process.
